# Value of incorporating newly identified risk factors into risk prediction for chemotherapy‐induced febrile neutropenia

**DOI:** 10.1002/cam4.1580

**Published:** 2018-06-28

**Authors:** Yanli Li, Leila Family, Lie H. Chen, John H. Page, Zandra Klippel, Lanfang Xu, Chun R. Chao

**Affiliations:** ^1^ Center for Observational Research Amgen Inc. South San Francisco CA USA; ^2^ Department of Research and Evaluation Kaiser Permanente Southern California Pasadena CA USA; ^3^ Center for Observational Research Amgen Inc. Thousand Oaks CA USA; ^4^ Clinical Development Amgen Inc. Thousand Oaks CA USA; ^5^ Medhealth Statistical Consulting Inc. Solon OH USA; ^6^ Present address: Leila Family, Los Angeles County Department of Public Health Office of Health Assessment and Epidemiology Los Angeles CA USA

**Keywords:** chemotherapy, comorbidities, febrile neutropenia, prediction model, risk factors

## Abstract

Several comorbidities have recently been shown to affect risk of chemotherapy‐induced febrile neutropenia (FN). Here, we evaluated the added predictive value of these comorbidities beyond established FN risk factors. A retrospective cohort study was conducted among adult patients diagnosed with cancer and treated with chemotherapy at Kaiser Permanente Southern California between 2000 and 2009. The study cohort was equally split into training and validation datasets to develop and evaluate the performance of FN risk prediction models in the first chemotherapy cycle. A reference model was developed based on the model proposed by Lyman et al (*Cancer* 2011;117:1917). A new model was developed by incorporating the newly identified comorbidities such as rheumatoid conditions and thyroid disorders into the reference model. Area under the receiver operating characteristic curve (AUROCC), risk reclassification, and integrated discrimination improvement (IDI) were used to evaluate the potential improvement of FN risk prediction by incorporating comorbidities. A total of 15 279 patients were included; 4.2% experienced FN in the first chemotherapy cycle. Including comorbidities in FN risk prediction did not improve AUROCC (reference model 0.71 vs new model 0.72). A significant improvement in individual‐level FN risk prediction was indicated by IDI (*P* = .02). However, significant improvement in risk reclassification was not observed overall (although 6% of all patients were more accurately classified for their FN risk level, 5% were less accurately classified) or when examining predicted FN risk among patients who did and did not develop FN. Incorporating several new comorbidities into FN prediction led to improved FN risk prediction in the first chemotherapy cycle, although the observed improvements were small and might not be clinically relevant.

## 1 INTRODUCTION

Chemotherapy‐induced febrile neutropenia (FN) is a clinically important adverse event that can negatively impact treatment outcomes. FN frequently requires hospitalization, which may result in significant healthcare costs.^1^, ^2^ Risk of developing FN is affected by chemotherapy regimens, patient characteristics, and disease characteristics. Clinical guidelines recommend primary prophylaxis with granulocyte colony‐stimulating factor (G‐CSF) in patients receiving high‐risk chemotherapy regimens (>20% risk in a chemotherapy course) and recommend consideration of G‐CSF prophylaxis for patients receiving intermediate‐risk chemotherapy regimens (10%‐20% risk in a chemotherapy course) who have additional risk factors.^3^, ^4^, ^5^, ^6^ Therefore, FN risk prediction tools incorporating patient and disease characteristics for individual patients could inform the clinical use of G‐CSF.

Lyman et al^7^ published an FN risk prediction model in 2011 (hereafter referred to as “the Lyman model”) that was based on prospectively collected data from community oncology practices in the USA. This model included risk factors such as patient age, cancer type, prior chemotherapy, laboratory measurements for abnormal hepatic and renal functions, low white blood cell (WBC) count, chemotherapy agents and planned relative dose intensity (RDI) ≥85%, concurrent immunosuppressive therapy, and receipt of G‐CSF prophylaxis as predictors for severe neutropenia or FN.^7^ This model has been validated in a study using electronic medical records (EMRs) from an external retrospective cohort of adult patients with cancer.^8^

Since the publication of the Lyman model,^7^ many comorbid conditions have been reported to be associated with risk of developing chemotherapy‐induced FN, including human immunodeficiency virus (HIV), diabetes (in nonoverweight patients), congestive heart failure, chronic obstructive pulmonary disease (COPD), autoimmune diseases, peptic ulcer disease, thyroid disease, liver disease, osteoarthritis, and recent dermatologic/mucosal conditions.^9^, ^10^, ^11^ The possible biologic mechanisms underlying these risk factors include bone marrow suppression, impaired neutrophil function, compromised skin/mucosal barrier integrity, and disturbances of the microbiome.^12^ Based on these hypothesized underlying pathological mechanisms, it is possible that these comorbidities may provide additional value in FN risk prediction. In this study, we evaluated the added predictive value of these comorbidities beyond the predictors identified in the Lyman model.^7^

## 2 MATERIALS AND METHODS

### 2.1 Study setting

This study was conducted at Kaiser Permanente Southern California (KPSC), an integrated managed care organization that provides comprehensive health services for 4 million enrollees with diverse race/ethnicity and socioeconomic backgrounds who broadly represent the residents of Southern California.^13^ KPSC maintains a number of EMRs for most aspects of care delivered, including diagnoses (eg, International Classification of Diseases [ICD]‐9 codes), medical procedures, pharmacy dispensing, laboratory test results, and disease registries. These EMRs are linkable through unique member identifiers. Incident cancer cases are recorded in KPSC's Surveillance, Epidemiology, and End Results (SEER)–affiliated cancer registry. All data for this study were collected using KPSC's EMRs and cancer registry.

### 2.2 Study design and patient selection

This study included adult patients (≥18 years of age) diagnosed with non‐Hodgkin's lymphoma (NHL), or breast, lung, colorectal, ovarian, or gastric cancer at KPSC between 2000 and 2009 and treated with chemotherapy within 12 months of cancer diagnosis and before December 2010. Patients who received prophylactic G‐CSF or prophylactic antibiotics were excluded as receipt of these medications would potentially alter FN risk in these patients. Patients were also excluded if they had <12 months of KPSC membership prior to cancer diagnosis (to allow the proper assessment of comorbidity status); had missing information on cancer stage or chemotherapy agents; received dose‐dense chemotherapy or weekly chemotherapy regimens; or had received a bone marrow or stem cell transplant.

The protocol for this study was approved by KPSC's Institutional Review Board. Formal informed consent was not required as the article reports results from a retrospective analysis of data in KPSC's EMRs and cancer registry and does not contain any studies involving direct contact of human participants performed by any of the authors.

### 2.3 Endpoint assessment

The endpoint of interest was FN in the first chemotherapy cycle. Only the first cycle was assessed to obtain the most unbiased FN risk, because FN risk in subsequent cycles might be affected by dose modification due to other complications. FN was defined by a combination of ICD‐9 codes, laboratory values, and health service utilization, using one of the following methods:^10^, ^11^, ^12^, ^14^ (1) neutropenia ICD‐9 code 288.0 and fever ICD‐9 code 780.6 (within 7 days); or (2) absolute neutrophil count (ANC) <1000/μL and fever ICD‐9 code 780.6 (within 7 days); or (3) hospitalization with neutropenia ICD‐9 code 288.0 as the primary diagnosis; or (4) neutropenia ICD‐9 code 288.0 or ANC <1000/μL within 7 days of hospitalization with ICD‐9 code of bacterial/fungal infection.

### 2.4 Assessment of exposure to FN risk factors

Predictors used in the Lyman model^7^ included patient's age, prior chemotherapy, serum aspartate aminotransferase (AST) level, serum alkaline phosphatase (AP) level, serum bilirubin level, glomerular filtration rate (GFR), WBC count, cancer type, immunosuppressive drug use, RDI, and type of chemotherapy agents. The status of each of these predictors was assessed for all eligible patients included in this study. The use of immunosuppressive drugs (see the list in Table S1) was defined as use for 2 weeks or longer within 3 months prior to chemotherapy. Chemotherapy agents and the percentage of planned dose received were assessed for the first chemotherapy cycle only. The percentage of planned dose received was calculated as average of [actual dose in the first chemotherapy cycle/standard dose in the first chemotherapy cycle] for all myelosuppressive agents. This is different from [actual dose in a chemotherapy course/standard dose in a chemotherapy course], the standard method used for determining the RDI of a given regimen. This change was necessitated by our focus in this study on the first chemotherapy cycle. Laboratory measurements within 6 months prior to chemotherapy initiation were identified. If multiple measurements were available for a given laboratory test, the value closest to chemotherapy initiation was used in the analysis.

The status of comorbidities that have been shown to be associated with FN risk was assessed (see Table S2 for methods for identifying comorbid conditions). HIV and diabetes were assessed using data from KPSC's disease registries. For HIV, all available data up to the time of cancer diagnosis were assessed. For diabetes, data from the earliest diagnosis date defined in the case identification algorithm from KPSC's case management system up to the time of cancer diagnosis were assessed. The history of selected dermatological/mucosal conditions was assessed within 1 month before chemotherapy initiation using ICD‐9 codes (Table S2). The presence of congestive heart failure, COPD, liver disease, osteoarthritis, rheumatoid disease, other autoimmune diseases (including inflammatory bowel disease, systemic lupus erythematosus, and multiple sclerosis), thyroid disorder, and peptic ulcer disease was assessed within the 12 months before chemotherapy initiation using ICD‐9 diagnosis codes (Table S2). Overweight/obesity was assessed using patients’ weight and height information recorded in the chemotherapy administration database at chemotherapy initiation. Overweight was defined as a body mass index (BMI) between 25.0 and <30.0 kg/m^2^ and obesity as a BMI of 30 kg/m^2^ or higher.^15^

### 2.5 Model development and evaluation

#### 2.5.1 Training and validation datasets

To evaluate the added predictive value of comorbidities for FN risk in the first chemotherapy cycle, the study population was randomly split into a training dataset and a validation dataset (in a 1:1 ratio) to lessen the impact of potential overfitting on evaluating risk prediction and assess the performance of the model. The training dataset was used to develop the new FN risk prediction model incorporating comorbidities, whereas the validation dataset was used to evaluate the performance of this new model in predicting FN risk, using the regression coefficients derived from the training dataset.

#### 2.5.2 Development of the reference model

**Figure 1:**
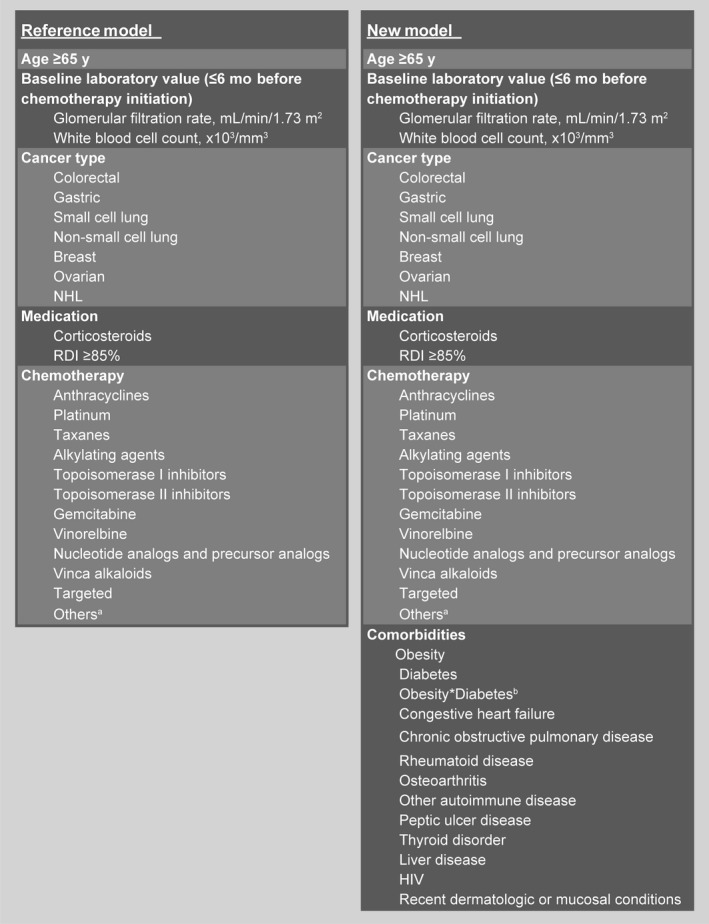
Predictors of febrile neutropenia risk in the first chemotherapy cycle included in the reference model and new risk factor model. ^a^Includes DNA cross‐linkers, epothilones, and immunomodulators. ^b^Interaction term between obesity and diabetes. HIV, human immunodeficiency virus; NHL, non‐Hodgkin's lymphoma; RDI, relative dose intensity

A reference model was developed based on the Lyman model^7^ with several modifications (Figure 1). Prior history of chemotherapy was omitted, because the complete history of chemotherapy might not be accurately assessed due to lack of patients’ information prior to KPSC enrollment. AST, AP, and bilirubin were omitted, because these were not routinely measured for all patients (missing > 10%), and patients who had these tests performed were more likely to have some clinical indications than those who did not. Several categories of chemotherapy agents (ie, nucleotide and precursor analogs, vinca alkaloids, targeted therapy, and others such as DNA cross‐linkers, epothilones, and immunomodulators) were added to account for the diverse chemotherapy agents used in this study. Additionally, the predictor of “G‐CSF prophylaxis” was not included in the reference model, because patients receiving G‐CSF primary prophylaxis were excluded from our study. Multivariate logistic regression model was used to derive odds ratios (ORs) and 95% confidence intervals (CIs) for the risk of developing FN in the first chemotherapy cycle in the training dataset.

#### 2.5.3 Development of the new prediction model

A new model with all the newly identified comorbidities of interest plus the predictors in the reference model was developed (Figure 1). A 2‐way interaction term between obesity and diabetes was included in the new model, as a previous study^12^ had shown that diabetes was associated with increased FN risk only among patients who were not overweight/obese.

#### 2.5.4 Evaluating added predictive value of comorbidities

The added predictive value of the comorbidities was assessed by comparing the performance of the reference model to that of the new model in the validation dataset. To compare the discriminative ability of the model, area under the receiver operating characteristic curve (AUROCC) was calculated for the reference model and the new model. The receiver operating characteristic curve is a plot of a model's sensitivity against 1 minus specificity. Area under the curve is a metric used to summarize the ability of a model to discriminate patients who developed an event of interest from those who did not, with an AUROCC of 1 corresponding to perfect discrimination and an AUROCC of 0.5 corresponding to no discrimination.

We also used risk reclassification tables to evaluate calibration in risk prediction improvement.^16^ First, an FN risk reclassification table for all patients in the validation dataset was generated by cross‐tabulating the FN risk categories in the first chemotherapy cycle predicted by the reference model and the new model. Risk reclassification is defined as improvement when the observed FN risk matches the new model's predicted risk category but not the reference model's and is defined as worsening when the observed FN risk matches the reference model's predicted risk category but not the new model's. Next, the risk reclassification tables were built separately for patients who did and did not develop FN. For patients who developed FN, any movement in the predicted risk to a higher FN risk category in the new model was considered improvement in risk prediction, whereas any movement to a lower FN risk category in the new model was considered worse reclassification. The opposite rule was applied for patients who did not develop FN. The improvement in predicted risk reclassification overall was quantified using net reclassification improvement (NRI).^16^ The following FN risk categories were considered in the reclassification analysis described above: <5%, 5% to <10%, and ≥10%. These ranges were chosen as it has been reported that approximately half of FN events occur in the first chemotherapy cycle;^3^, ^17^ therefore, they are likely equivalent to approximately <10%, 10% to 20%, and >20% FN risk over the chemotherapy course, which are cutoffs used in clinical guidelines.^3^, ^4^, ^5^, ^6^

The integrated discrimination improvement (IDI), a composite measurement of the increment in the predicted risk for patients with FN and the reduction of predicted risk for the patients without FN, was also calculated to assess the model performance independent of the choice of risk category cutoffs.^18^, ^19^

### 2.6 Sensitivity analysis

A sensitivity analysis was performed by creating a reference model that included all the predictors that had been used in the original Lyman model (except for G‐CSF prophylaxis) among a subcohort that had complete data for all the relevant laboratory predictors (ie, including AST, AP, bilirubin, WBC, and GFR). We used the history of prior cancer as a proxy for prior chemotherapy in this sensitivity analysis (of note, the history of prior cancer could also be incomplete due to lack of patients’ information for time periods prior to KPSC enrollment). Performance of the new model was then assessed in the subset that had complete data.

## 3 RESULTS

### 3.1 Patients

**Figure 2:**
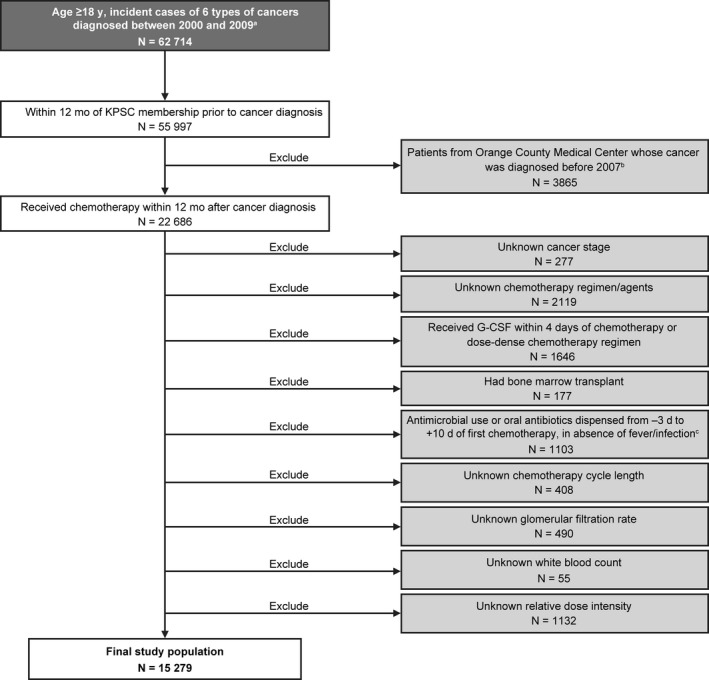
Patient selection. ^a^The 6 tumor types include non‐Hodgkin's lymphoma, and breast, lung, colorectal, ovarian, and gastric cancers. ^b^Excluded due to lack of radiation therapy data. ^c^Antimicrobial use includes the use of any prophylactic antibacterial, antifungal, or antiviral agent. G‐CSF, granulocyte colony‐stimulating factor; KPSC, Kaiser Permanente Southern California

The analysis included 15 279 patients (Figure 2). Of these, 69.0% were female, and 38.3% were ≥65 years of age, with mean age at cancer diagnosis of 60.2 years (Table 1). Primary tumor types included NHL (10.3%) and breast (38.4%), lung (22.8%), colorectal (19.7%), ovarian (5.8%), and gastric cancers (3.0%). FN incidence in the first chemotherapy cycle was 4.2%. Similar distribution of demographic and treatment characteristics was observed in the training and validation datasets (Table 1).

**Table 1:** Patient demographic and treatment characteristics

Characteristics	Total (N =* *15 279)	Training dataset (N =* *7640)	Validation dataset (N =* *7639)
Female, n (%)	10 543 (69.0)	5260 (68.9)	5283 (69.2)
Age at cancer diagnosis, mean (SD), years	60.2 (11.9)	60.2 (12.0)	60.2 (11.8)
Age at cancer diagnosis ≥65 yr, n (%)	5859 (38.3)	2932 (38.4)	2927 (38.3)
Baseline laboratory value^a^			
Glomerular filtration rate, mean (SD), mL/min/1.73 m^2^	80.5 (24.82)	80.6 (26.00)	80.3 (23.57)
White blood cell count, mean (SD), ×10^3^/mm^3^	8.1 (4.55)	8.1 (4.17)	8.1 (4.91)
Cancer type, n (%)			
Breast	5867 (38.4)	2898 (37.9)	2969 (38.9)
Colorectal	3014 (19.7)	1509 (19.8)	1505 (19.7)
Non‐small cell lung	2747 (18.0)	1339 (17.5)	1408 (18.4)
NHL	1573 (10.3)	804 (10.5)	769 (10.1)
Ovarian	890 (5.8)	466 (6.1)	424 (5.5)
Small cell lung	727 (4.8)	383 (5.0)	344 (4.5)
Gastric	461 (3.0)	241 (3.2)	220 (2.9)
Medication			
Immunosuppressive drugs,^b^ n (%)	1381 (9.0)	659 (8.6)	722 (9.5)
Planned RDI ≥85%,^c^ n (%)	13 174 (86.2)	6613 (86.6)	6561 (85.9)
Chemotherapy, n (%)			
Alkylating agents	6627 (43.4)	3295 (43.1)	3332 (43.6)
Platinum	6003 (39.3)	3032 (39.7)	2971 (38.9)
Anthracyclines	5446 (35.6)	2698 (35.3)	2748 (36.0)
Nucleotide analogs and precursor analogs	4784 (31.3)	2413 (31.6)	2371 (31.0)
Taxanes	4175 (27.3)	2089 (27.3)	2086 (27.3)
Targeted	1830 (12)	929 (12.2)	901 (11.8)
Vinca alkaloids	1418 (9.3)	724 (9.5)	694 (9.1)
Topoisomerase II inhibitors	861 (5.6)	460 (6)	401 (5.2)
Gemcitabine	521 (3.4)	260 (3.4)	261 (3.4)
Topoisomerase I inhibitors	442 (2.9)	224 (2.9)	218 (2.9)
Vinorelbine	113 (0.7)	37 (0.5)	76 (1.0)
Others (DNA cross‐linkers, epothilones, and immunomodulators)	40 (0.3)	27 (0.4)	13 (0.2)
Comorbidities,^d^^,^ e n (%)			
Obesity	4257 (27.9)	2062 (27.0)	2195 (28.7)
Chronic obstructive pulmonary disease	3007 (19.7)	1506 (19.7)	1501 (19.6)
Diabetes	2676 (17.5)	1334 (17.5)	1342 (17.6)
Osteoarthritis	1890 (12.4)	921 (12.1)	969 (12.7)
Thyroid disorder	1584 (10.4)	800 (10.5)	784 (10.3)
Congestive heart failure	558 (3.7)	284 (3.7)	274 (3.6)
Peptic ulcer disease	394 (2.6)	192 (2.5)	202 (2.6)
Rheumatoid disease	280 (1.8)	137 (1.8)	143 (1.9)
Liver disease	261 (1.7)	138 (1.8)	123 (1.6)
Other autoimmune disease^f^	126 (0.8)	67 (0.9)	59 (0.8)
HIV	53 (0.3)	28 (0.4)	25 (0.3)
Recent dermatologic or mucosal conditions,^d^^,^ g n (%)	455 (3)	223 (2.9)	232 (3)

HIV, human immunodeficiency virus; NHL, non‐Hodgkin's lymphoma; SD, standard deviation; RDI, relative dose intensity.

a ≤6 mo before chemotherapy initiation.

b ≥2‐wk use within 3 mo prior to chemotherapy initiation.

c Based on the first chemotherapy only, using the average (actual dose/standard dose) of all myelosuppressive drugs.

d Newly identified risk/protective factors.

e Assessed within 12 mo before chemotherapy initiation.

f Includes inflammatory bowel disease, systemic lupus erythematosus, and multiple sclerosis.

g ≤1 mo before chemotherapy initiation.

### 3.2 Model derivation

The new model included the predictors in the reference model plus all comorbidities reported to be associated with FN risk in the literature, including HIV, diabetes, congestive heart failure, COPD, rheumatoid diseases, other autoimmune diseases, peptic ulcer disease, thyroid disease, liver disease, osteoarthritis, recent dermatologic/mucosal conditions, overweight/obesity, and an interaction term between diabetes and overweight/obesity (Figure 1). Table 2 summarizes the predictors that were included in the reference model and the new model, and the ORs and 95% CIs for the risk of developing FN in the first chemotherapy cycle in the training dataset.

**Table 2:** Effect of predictors included in the reference model and new risk factor model on FN risk in the first chemotherapy cycle derived using the training dataset

Predictor	Reference model Odds ratio (95% CI)	New model Odds ratio (95% CI)
Age ≥65 yr	1.16 (0.90, 1.48)	1.06 (0.82, 1.37)
Baseline laboratory value^a^		
Glomerular filtration rate, mL/min/1.73 m^2^	1.00 (0.99, 1.00)	1.00 (0.99, 1.00)
White blood cell count, ×10^3^/mm^3^	1.03 (1.01, 1.04)	1.03 (1.01, 1.05)
Cancer type		
Colorectal	1.00	1.00
Gastric	3.66 (1.81, 7.43)	3.18 (1.54, 6.56)
Small cell lung	2.14 (0.94, 4.87)	1.90 (0.83, 4.36)
Non‐small cell lung	0.62 (0.29, 1.33)	0.62 (0.29, 1.33)
Breast	2.00 (0.93, 4.29)	2.02 (0.94, 4.35)
Ovarian	0.42 (0.17, 1.02)	0.41 (0.17, 1.01)
NHL	4.38 (1.59, 12.07)	3.90 (1.39, 10.90)
Medication		
Immunosuppressive drugs^b^	1.40 (1.01, 1.94)	1.28 (0.92, 1.80)
RDI ≥ 85%^c^	1.37 (0.97, 1.94)	1.40 (0.99, 1.98)
Chemotherapy		
Anthracyclines	1.37 (0.95, 1.98)	1.41 (0.97, 2.06)
Platinum	0.97 (0.63, 1.49)	0.95 (0.62, 1.47)
Taxanes	4.79 (3.15, 7.28)	4.68 (3.07, 7.12)
Alkylating agents	0.64 (0.40, 1.01)	0.62 (0.39, 0.97)
Topoisomerase I inhibitors	2.13 (0.97, 4.67)	2.15 (0.97, 4.73)
Topoisomerase II inhibitors	2.31 (1.33, 4.01)	2.32 (1.33, 4.04)
Gemcitabine	3.45 (1.75, 6.80)	3.40 (1.71, 6.76)
Vinorelbine	2.05 (0.27, 15.75)	1.90 (0.25, 14.63)
Nucleotide analogs and precursor analogs	0.70 (0.45, 1.09)	0.70 (0.45, 1.09)
Vinca alkaloids	1.65 (0.71, 3.83)	1.71 (0.73, 3.99)
Targeted	0.99 (0.66, 1.46)	0.99 (0.66, 1.47)
Others (DNA cross‐linkers, epothilones, and immunomodulators)	5.97 (2.08, 17.09)	5.94 (1.97, 17.89)
Comorbidities^d^^,^ e		
Obesity	Not included	0.94 (0.70, 1.27)
Diabetes	Not included	1.16 (0.82, 1.64)
Obesity*diabetes^f^	Not included	0.85 (0.47, 1.55)
Congestive heart failure	Not included	1.03 (0.61, 1.76)
Chronic obstructive pulmonary disease	Not included	1.22 (0.93, 1.61)
Rheumatoid disease	Not included	2.03 (1.15, 3.56)
Osteoarthritis	Not included	1.25 (0.92, 1.70)
Other autoimmune disease^g^	Not included	0.87 (0.26, 2.91)
Peptic ulcer disease	Not included	1.62 (0.94, 2.81)
Thyroid disorder	Not included	1.51 (1.10, 2.08)
Liver disease	Not included	1.73 (0.93, 3.22)
HIV	Not included	1.48 (0.41, 5.31)
Recent dermatologic or mucosal conditions^d^^,^ h	Not included	1.63 (1.01, 2.64)

CI, confidence interval; FN, febrile neutropenia; HIV, human immunodeficiency virus; NHL, non‐Hodgkin's lymphoma; RDI, relative dose intensity.

a ≤6 mo before chemotherapy initiation.

b ≥2‐wk use within 3 mo prior to chemotherapy initiation.

c Based on the first chemotherapy only, using the average (actual dose/standard dose) of all myelosuppressive drugs.

d Newly identified risk/protective factors.

e Assessed within 12 mo before chemotherapy initiation.

f Interaction term between obesity and diabetes.

g Includes inflammatory bowel disease, systemic lupus erythematosus, and multiple sclerosis.

h ≤1 mo before chemotherapy initiation.

### 3.3 Model performance

No improvement in AUROCC was observed in the new model (0.72) compared with the reference model (0.71). Adding comorbidities only led to slightly more patients being classified into correct FN risk categories. Table 3 cross‐tabulates predicted risk with the reference model, predicted risk with the new model, and observed FN risk. Patients more accurately classified by the new model are shown in green cells, while patients less accurately classified are shown in red cells. For the overall population, the new model provided more accurate risk classification for 6% of patients (n = 453; 218 + 235), as the observed FN risk matches the new model's predicted risk category (green cells) but not the reference model's predicted risk category. For example, the observed FN risk for the 235 patients is 4.7%, which matches the new model's predicted risk category of 0%‐5% but not the reference model's predicted risk category of 5% to <10%. The new model provided less accurate risk classification for 5% of patients (n = 390; 11 + 173 + 206) (red cells), as the observed FN risk matches the reference model's predicted risk category but not the new model's predicted risk category.

**Table 3:** Predicted FN risks by the reference model and new model compared to the observed risks in the first chemotherapy cycle

Reference model^a^	New model^a^			
	FN risk category			
FN risk category	<5%	5% to <10%	≥10%	Total
<5%				5376
n	5147	218	11	
Observed FN risk	2.1%	5.0%	0%	
5% to <10%				1512
n	235	1104	173	
Observed FN risk	4.7%	6.9%	9.8%	
≥10%				751
n	0	206	545	
Observed FN risk	NA	10.7%	13.0%	
Total	5382	1528	729	7639

FN, febrile neutropenia; NA, not applicable.

a Predictors included in the reference model and new model are shown in Figure 1 and Table 2.

Compared to the observed FN risks:

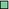
, Risk reclassification improved by the new risk factor model (n = 453 [218 + 235]).

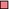
, Risk reclassification worsened by the new risk factor model (n = 390 [11 + 173 + 206]).

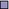
, Both new risk factor model and reference model classified patients into correct FN risk categories (n = 6769 [5147 + 1104 + 545]).

Table 4 summarizes risk reclassifications for patients who did and did not develop FN in the first chemotherapy cycle. Of the 317 patients in the validation dataset who developed FN, the new model improved classification for 8.8% of patients (n = 28 [17 + 11]) but worsened classification for 7.3% of patients (n = 33 [11 + 22]). Of the 7 322 patients who did not develop FN, the new model improved risk classification for 5.6% of patients (n = 408 [224 + 184]) but worsened risk classification for 5.1% of patients (n = 374 [207 + 11 + 156]). NRI, a metric used to quantify the overall improvement in risk reclassification among patients who developed and did not develop FN, was not statistically significant for the new model (*P* = .65) compared to the reference model.

**Table 4:** Risk reclassification among patients who developed FN vs those who did not develop FN in the first chemotherapy cycle in the new model

Reference model^a^	New model^a^			
	FN risk category			
FN risk category	<5%	5% to <10%	≥10%	Total
Patients who developed FN				
<5%				120
n	109	11	0	
5% to <10%				104
n	11	76	17	
≥10%				93
n	0	22	71	
Total	120	109	88	317
Patients who did not develop FN				
<5%				5256
n	5038	207	11	
5% to <10%				1408
n	224	1028	156	
≥10%				658
n	0	184	474	
Total	5262	1419	641	7322

FN, febrile neutropenia.

a Predictors included in the reference model and new model are shown in Figure 1 and Table 2.

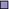
, No difference between the 2 models: patients with FN (n = 256 [109 + 76 + 71]) and patients without FN (n = 6540 [5038 + 1028 + 474]) were classified in the same risk categories by both models.

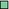
, New risk factor model improved risk classification: patients with FN reclassified into higher‐risk categories (n = 28 [11 + 17]), and patients without FN reclassified into lower‐risk categories (n = 408 [224 + 0 + 184]).

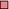
, New risk factor model worsened risk classification: patients with FN reclassified into lower‐risk categories (n = 33 [11 + 0 + 22]), and patients without FN reclassified into higher‐risk categories (n = 374 [207 + 11 + 156]).

Integrated discrimination improvement, a metric used to assess the model performance independent of the choice of risk category cutoffs, was found to be statistically significant for the new model (*P* = .02) compared to the reference model.

### 3.4 Sensitivity analysis

The sensitivity analysis included 10 046 patients with complete laboratory data. In the validation set (n = 5030), incorporating comorbidities into the Lyman model^7^ did not result in increase in AUROCC (from 0.70 to 0.70). The model with comorbidities improved predicted risk classification for 3% of patients (171 [98 + 73]) but worsened predicted risk classification for 5% of patients (n = 274 [145 + 128 + 1]) (Table S3). NRI (*P* = .911) and IDI (*P* = .65) were not statistically significant.

## 4 DISCUSSION

We found that including recently identified comorbidities that are associated with FN risk led to improved individual‐level FN risk prediction in the first chemotherapy cycle as measured by the IDI. However, the improvement we observed was small, and it may not translate into meaningful risk reclassification or an improvement in the clinical prophylactic management of FN.

Although clinical guidelines^3^, ^4^, ^5^, ^6^ recommend consideration of patient‐level risk factors when making G‐CSF prophylaxis decisions, it is not clear how prophylaxis should be best directed based on the presence of these patient‐level risk factors. A risk prediction model incorporating all relevant patient‐level risk factors is thus needed to guide prophylactic decisions. The intent of this study was to evaluate whether previously established FN risk prediction models should be updated to include newly identified FN risk factors. Such an update might have implications for clinical management of cancer patients undergoing chemotherapy. However, statistically significant risk factors may or may not be important contributors to risk prediction, depending on their distributions in the population.^20^ A previous study also suggested that for models containing standard risk factors and with reasonably good discrimination, very large “independent” associations of the new marker with the outcome are required to result in a meaningful increase in AUROCC.^16^ These considerations call for robust evaluations of the added predictive value of novel risk factors.

That said, the interpretation of the NRI and risk reclassification tables requires caution, as these measures depend on the choice of clinically meaningful cutoffs. We assumed that FN incidence in the first cycle is half the FN incidence over the whole course of chemotherapy. Although this appears true in some cohorts,^17^ FN distribution across cycles depends on the chemotherapy regimen as well as how many cycles are present in the regimen. If the true FN incidence proportion in the first cycle is not half of that over the chemotherapy course, our statistical methods remain valid, but the statistics for NRI and the numbers in Tables 3 and 4 would be different; IDI would remain the same, as it is independent of choice of cutoffs. Further, both clinical trials and observational studies are subject to selection bias due to differential censoring. For example, patients who developed other severe adverse events may die, may need to switch regimens, or may terminate treatment early. As a result, patients who complete the entire course of treatment are likely different than the entire patient cohort at the beginning of chemotherapy. The FN risk cutoffs chosen based on the available data thus might not reflect the appropriate cutoffs.

In our reference model, we expanded the chemotherapy classes beyond what was included in the Lyman model,^7^ to fully account for the chemotherapy agents used in the study population. In addition to alkylating agents, platinum, anthracyclines, taxanes, topoisomerase I and II inhibitors, gemcitabine, and vinorelbine included in the Lyman model^7^ (Figure 1), we also included nucleotide and precursor analogs, targeted therapy, vinca alkaloids, and others. This expansion to the reference model was not subject to any form of model selection; therefore, it is possible that some of the newly added chemotherapy drug classes may have no effect on FN risk as suggested by the OR estimates and could be omitted from the prediction model. However, because our purpose was to evaluate the incremental predictive value of adding comorbidities to established risk factors, we included more chemotherapy drug classes to ensure that the effects of chemotherapy on FN were appropriately captured. Of note, we found that chemotherapy agents that were classified into the “others” category appeared to be associated with highly elevated FN risk (OR = 5.97, 95% CI: 2.08, 17.09) (Table 2), independent of other predictors in the reference model as well as the new model. Agents in this category, including DNA cross‐linkers, epothilones, and immunomodulators, were only used by 0.3% of the study population. Further studies with large sample sizes are needed to better understand the impact of these agents on FN risk.

Several limitations should be considered when interpreting the results of our study. In addition to those mentioned above, the retrospective use of EMR data is subject to potential misclassification of the FN outcome and predictors due to inadequate or undercoding, which may negatively affect the evaluation of model performance. We developed and evaluated the new prediction models in the same study population that was previously used to identify these comorbidities as FN risk factors;^9^, ^10^, ^11^, ^12^ however, we split the study population into training and validation datasets and applied the beta coefficients obtained in the training set to the validation set to mitigate potential issues with overfitting. Patients who were excluded from the study because they received G‐CSF or antibiotics might have been those at highest risk of FN or those with comorbidities, and this may have left lower‐risk patients in the study cohort and biased our results. However, the impact of this exclusion is likely only moderate as only 7% of those eligible were excluded due to prophylactic G‐CSF use or receipt of a dose‐dense chemotherapy regimen. Several predictors in the Lyman model^7^ were omitted from the reference model in this study to avoid performing model development and evaluation in a biased sample. We also made the assumption that incidence of FN in the first cycle is half of that over the whole course of chemotherapy to derive NRI. This assumption might not be valid, which might partially explain the inconsistent findings measured by NRI and IDI.

In conclusion, incorporating several new comorbidities into established FN risk factors led to improved FN risk prediction in the first chemotherapy cycle in the patient population from a large community‐based practice, although the observed improvements were small and might not be clinically relevant. The best FN risk prediction model should be continually evaluated as knowledge on new FN risk factors becomes available.

## DISCLOSURE STATEMENT

This study was funded by Amgen Inc. Yanli Li, John H. Page, and Zandra Klippel report employment by and hold shares in Amgen Inc. Leila Family reports employment by Kaiser Permanente Southern California (KPSC) at the time of the study; she is currently employed by the Los Angeles County Department of Public Health, Office of Health Assessment and Epidemiology. Lie Hong Chen and Chun R. Chao report employment by KPSC, which received research funding for this study from Amgen Inc. Lanfang Xu reports employment by Medhealth Statistical Consulting Inc. and being an independent contractor for KPSC, which received research funding for this study from Amgen Inc.
