# Supplementary material for: Value of incorporating newly identified risk factors into risk prediction for chemotherapy‐induced febrile neutropenia
Source: Cancer Med. 2018 Jun 28;7(8):4121–31. doi: 10.1002/cam4.1580 (PMC6089155; doi:10.1002/cam4.1580)
Supplement: Supplementary file 1 [file CAM4-7-4121-s001.pdf]

APPENDIX SECTION

Value of incorporating newly identified risk factors into risk prediction for chemotherapy-induced febrile neutropenia

Authors: Yanli Li,[1] Leila Family,[2,*] Lie Hong Chen,[2] John H. Page,[3] Zandra Klippel,[4] Lanfang Xu,[5] Chun R. Chao[2]

Affiliations:

[1]Center for Observational Research, Amgen Inc., 1150 Veterans Blvd, South San Francisco, California 94080, USA

[2]Department of Research and Evaluation, Kaiser Permanente Southern California, 100 S Los Robles Ave, Pasadena, California 91101, USA

[3]Center for Observational Research, Amgen Inc., 1 Amgen Center Drive, Thousand Oaks, California 91320, USA

[4]Clinical Development, Amgen Inc., 1 Amgen Center Drive, Thousand Oaks, California 91320, USA

[5]Medhealth Statistical Consulting Inc., 6848 Silkwood Ln, Solon, Ohio 44139, USA

*Currently at the Los Angeles County Department of Public Health, Office of Health Assessment and Epidemiology, 313 N. Figueroa St., Los Angeles, California 90012, USA

Running head: New risk factors for FN prediction

Corresponding author:

Chun R. Chao, PhD; Department of Research and Evaluation; Kaiser Permanente Southern California; 100 S Los Robles Ave; Pasadena, CA 91101, USA

Phone: +1 626-564-3797; Fax: +1 626-564-3797; Email: Chun.R.Chao@kp.org

List of Tables in Appendix Section

Appendix Table 1. List of Immunosuppressive Drugs

Appendix Table 2. Methods for Identifying Comorbid Conditions

Appendix Table 3. Predicted FN Risks by the Reference Model and New Model Compared to the Observed Risks in the First Chemotherapy Cycle in the Sensitivity Analysis

Appendix Table 1. List of Immunosuppressive Drugs

Drug
Azathioprine
Betamethasone
Budesonide
Cortisone
Cyclosporin
Dexamethasone
Fludrocortisone
Hydrocortisone
Methylprednisolone
Mycophenolate
Paramethasone acetate
Prednisolone
Prednisone
Sirolimus
Tacrolimus
Triamcinolone

Appendix Table 2. Methods for Identifying Comorbid Conditions

Condition	ICD-9 Diagnosis Code[a]	Disease Registry/Chemotherapy Database
Congestive heart failure	428	
COPD/Emphysema	490–496, 500–505, 506.4	
Dermatologic or mucosal conditions	690–694, 696, 698, 701, 707, 708	
Diabetes		KPSC case management system identification criteria<br><u>Inclusion criteria:</u><br>Age >18 years and a current KPSC member and<br>Any discharge hospital ICD-9 code of 250.xx, 357.2, 362.0, 366.41, or 648.0 or<br>Any ICD-9 code of 250.xx, 357.2, 362.0, 366.41, or 648.0 or<br>Any KPSC internal code for diabetes-related diagnosis: '1200', '1201', '1202', '1203', '1204', '1839', '3141', '3186', '3639', '4124', '5782' or<br>HbA1c > 7.5% or Fructosamine > 319 µmol/L or<br>A dispensing record for an oral hypoglycemic or insulin or<br>Placed on the diabetes-patient-addition list<br><u>Exclusion criteria:</u><br>Member deceased or<br>Female member coded for polycystic ovarian syndrome and identified by metformin only or<br>Member coded for gestational diabetes or<br>Female member with a positive pregnancy test is temporarily excluded from the diabetes population for 9 months after the positive pregnancy test date (member will return to the diabetes population if the above inclusion criteria

		is dated after the 9-month period or placed on the case management diabetes-patient-deletion list.
HIV infection		HIV registry
Liver disease	571–572, 456.0–456.21	
Osteoarthritis	715	
Other autoimmune conditions[b]	555, 695.4, 710.0, 340	
Peptic ulcer disease	531–534	
Rheumatoid disease[c]	714, 725	
Thyroid disorder (including autoimmune thyroiditis)	240–246	

[a]One inpatient ICD-9 code or 2 outpatient ICD-9 codes on separate dates were required to define the presence of the comorbidity.
[b]Other autoimmune conditions include inflammatory bowel disease, systemic lupus erythematosus, and multiple sclerosis.
[c]Rheumatoid disease includes rheumatoid arthritis and polymyalgia rheumatica.
COPD, chronic obstructive pulmonary disease; HbA1c, glycated hemoglobin; HIV, human immunodeficiency virus; ICD, International Classification of Diseases; KPSC, Kaiser Permanente Southern California.

Appendix Table 3. Predicted FN Risks by the Reference Model and the New Model Compared to the Observed Risks in the First Chemotherapy Cycle in the Sensitivity Analysis

**Reference Model[a]**	**New Model[a]**			
	**FN Risk Category**			
**FN Risk Category**	<5%	5% to <10%	≥10%	Total
<5%				3577
n	3448	128	1	
Observed FN risk	*2.3%*	*4.7%*	*0%*	
5% to <10%				993
n	145	750	98	
Observed FN risk	*7.6%*	*6.1%*	*11.2%*	
≥10%				460
n	0	73	387	
Observed FN risk	*NA*	*6.8%*	*13.7%*	
Total	3593	951	486	5030

Compared to the observed FN risks:
- (green) Risk reclassification improved by the new risk factors model (n=171 [98+73]).
- (red) Risk reclassification worsened by the new risk factors model (n=274 [145+128+1]).
- (white) Both new risk factors model and reference model classified patients into correct FN risk categories (n=4585 [3448+750+387]).

[a]Predictors included in the reference model and new model as shown in Figure 1 and Table 2 plus AST, AP, bilirubin, and prior cancer as a proxy for prior chemotherapy.
AP = alkaline phosphatase; AST = aspartate aminotransferase; FN, febrile neutropenia; NA = not applicable.
